# Clinical Study on Prospective Efficacy of All-Trans Acid, Realgar-Indigo Naturalis Formula Combined with Chemotherapy as Maintenance Treatment of Acute Promyelocytic Leukemia

**DOI:** 10.1155/2014/987560

**Published:** 2014-05-20

**Authors:** Li Xiang-Xin, Wang Lu-Qun, Li Hao, He Xiao-Peng, Li Fang-Lin, Wang Ling-Ling, Chen Xue-Liang, Hou Ming

**Affiliations:** ^1^Department of Haematology, QiLu Hospital of Shandong University, Jinan, Shandong 250012, China; ^2^Department of Thoracic Surgery, Provincial Hospital Affiliated to Shandong University, Jinan, Shandong 250021, China

## Abstract

*Objectives*. To test the efficiency and safety of sequential application of retinoic acid (ATRA), Realgar-Indigo naturalis formula (RIF) and chemotherapy (CT) were used as the maintenance treatment in patients with acute promyelocytic leukemia (APL). *Methods*. This was a retrospective study of 98 patients with newly diagnosed APL who accepted two different maintenance treatments. After remission induction and consolidation chemotherapy according to their Sanz scores, patients received two different kinds of maintenance scheme. The first regimen was using ATRA, RIF, and standard dose of CT sequentially (ATRA/RIF/CT regimen), while the second one was using ATRA and low dose of chemotherapy with methotrexate (MTX) plus 6-mercaptopurine (6-MP) alternately (ATRA/CT_low_ regimen). The OS, DFS, relapse rate, minimal residual disease, and adverse reactions in two groups were monitored and evaluated. *Results*. ATRA/RIF/CT regimen could effectively reduce the chance of relapse in different risk stratification of patients, but there was no significant difference in 5-year DFS rate and OS rate between the two groups. Besides, the patients in the experimental group suffered less severe adverse reactions than those in the control group. *Conclusions*. The repeated sequential therapeutic regimen to APL with ATRA, RIF, and chemotherapy is worth popularizing for its high effectiveness and low toxicity.

## 1. Introduction

At present, acute promyelocytic leukemia (APL) has changed from a highly fatal disease to a curable disease. Therefore, the pursuit of treating APL is to minimize recurrence rate and achieve long-term survival. The widely accepted postremission therapy of APL is 2-3 cycles of anthracycline containing chemotherapy following retinoic acid (ATRA) and small dose of chemotherapy with methotrexate (MTX) and 6-mercaptopurine (6-MP) as maintenance therapy to 2 years [1]. Yet, LPA99 research [2] showed that 5-year cumulative relapse rates were 3%, 8%, and 25% in low, intermediate, and high risk patients, respectively, which showed that this scheme was not ideal as maintenance treatment, especially in high-risk group. The reason may be related to ATRA resistance, decreased medicine adherence to 6-MP, and lack of effective way to eliminate leukemic stem cells, which is the unique role of arsenic in treating APL [3]. Realgar-Indigo naturalis formula (RIF) is a representative oral arsenical formula which has got breakthrough progress on explaining its unique mechanism in treating APL with analytical and synthetic research approaches. The research of Wang et al. [4] revealed that the major components were As4S4, indirubin, and tanshinone. As4S4 causes intensified degradation of PML/RAR*α*, and tanshinone significantly promotes the expression of cell differentiation genes, while indirubin significantly inhibits cell cycle. In addition, tanshinone and indirubin increase the expression of aquaglyceroporin 9 and facilitate the transportation of As4S4 into APL cells, which in turn enhance As4S4 mediated PML-RAR*α* degradation and strengthen therapeutic efficiency. Therefore, different from arsenic trioxide (ATO), whose mechanism is inducing apoptosis only, RIF could make synergistic effect of three components and strengthen the antileukemia effect of arsenic. The effect of RIF has been confirmed in vitro and in murine APL model in vivo [5]. A multicenter phase II and phase III clinical trial in China [6] also showed that the effectiveness of this formula is similar to or even better than ATO with reasonable safety. Another advantage is its oral administration, which can reduce hospitalization cost and trauma. So, RIF is being gradually recognized and accepted in the world.

The good curative effect in the treatment of APL shows that arsenic inducing apoptosis, arsenic retinoic acid inducing cell differentiation, and chemotherapy drug inducing cytotoxic killing effects can provide different effects and play complementary roles. To minimize recurrence, we designed a maintenance treatment schedule, in which the patients accepted sequential application of retinoic acid, RIF, and chemotherapy with interval lengthened by year after 3 cycles of consolidation chemotherapy. Based on this scheme, the information of those who took this scheme and those who accepted traditional ATRA/CT_low_ regimen during that period has been analyzed retrospectively. From observing long-term survival, recurrence, and side effects of two groups, we attempt to explore the feasibility of this scheme as the maintenance treatment.

## 2. Materials and Methods

### 2.1. Patients

This was a retrospective study of 98 patients with newly diagnosed APL from January 2003 to December 2008 in QiLu Hospital of Shandong University. The diagnosis was based on morphology (promyelocytes on peripheral smear and/or bone marrow) and cytogenetic analysis of t(15; 17) chromosomal translocation and/or PML-RAR*α* gene detection by Q-PCR. Eligibility criteria included age from 13 to 65 years, adequate hepatic and renal reserve (defined as total bilirubin, ALT, AST, and creatinine ≤ 2.0 × the institutional upper limit of normal), and a WHO performance status score of 2 or lower. All participants signed written informed consent in accordance with the Declaration of Helsinki.

### 2.2. Remission Induction and Consolidation Chemotherapy

All patients accepted remission induction and consolidation chemotherapy according to their Sanz score [7], which is low-risk (WBC count ≤ 10 × 10^9^/L and platelet count > 40 × 10^9^/L), intermediate-risk (WBC count ≤ 10 × 10^9^/L and platelet count ≤ 40 × 10^9^/L), and high-risk (WBC count > 10 × 10^9^/L and platelet count ≤ 40 × 10^9^/L). The low-risk patients received ATRA (45 mg/m^2^ per day) alone until hematological CR. The intermediate-risk patients received ATRA (45 mg/m^2^ per day) plus daunorubicin (DNR, 40 mg/m^2^ per day for 3 days) and would adopt DA regimen (DNR 40 mg/m^2^ per day for 3 days and Ara-c 150 mg/m^2^ per day for 7 days) if insufficient chemotherapy as DNR was used alone. The high-risk patients received associated chemotherapy, using a combination of DA regimen with ATRA (45 mg/m^2^ per day) and ATO (0.16 mg/m^2^) until CR.

After achievement of CR1, patients received three courses of sequential consolidation chemotherapy with DA regimen (DNR 40 mg/m^2^ per day for 3 days and Ara-c 150 mg/m^2^ per day for 7 days), MA regimen (mitoxantrone 8–10 mg/m^2^ per day for 3 days and Ara-c 150 mg/m^2^ per day for 7 days), and HA regimen (homoharringtonine 2 mg/m^2^ per day for 7 days and Ara-c 150 mg/m^2^ per day for 7 days). After consolidation chemotherapy, they received maintenance chemotherapy.

### 2.3. Study Design in Maintenance Treatment

**Figure 1:**
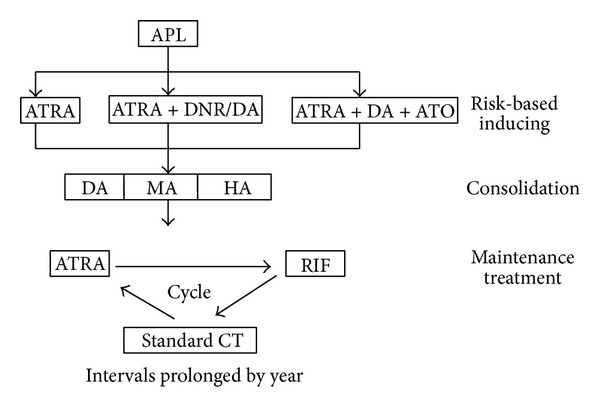
Treatment schedule in study group. ATRA: all-trans retinoic acid; ATO: arsenic trioxide; RIF: Realgar-Indigo naturalis formula; DNR: daunorubicin; MIT: mitoxantrone; HHT: homoharringtonine; Ara-c: cytarabine; CT: chemotherapy.

In maintenance treatment, 52 patients sequentially took RIF (60 mg/kg·d) for one month, ATRA (45 mg/m^2^) for one month, and standard chemotherapy (in which DA, MA, and HA regimens were used sequentially), with an interval of one month between each regimen in the first year and an interval of two months in the second year. In this ATRA/RIF/CT regimen, RIF (270 mg per pill) was provided by the Anhui Tiankang Group Pharmaceutical Resin Company, Tianchang, Anhui, China. RIF contained Realgar- (30 mg per pill), Indigo naturalis (125 mg per pill), Radix Salviae Miltiorrhizae (50 mg per pill), Radix Pseudostellariae (45 mg per pill), and garment film (20 mg per pill). The other control group included 46 patients who accepted ATRA and low dose chemotherapy (ATRA/CT_low_) regimen during the same period. The proposal in this group was ATRA 45 mg/m^2^ per day, three times per day for 15 days every 3 months combined with MTX 30 mg per week and 6-MP 50 mg, twice per day. All maintenance therapies lasted for two years (Figure 1).

After compression, all patients received lumbar puncture and intrathecal injection with MTX (10 mg) and Ara-c (50 mg) plus dexamethasone (5 mg) for four times in order to prevent CNS leukemia.

### 2.4. Minimal Residual Disease Monitoring and Toxicity Criteria

PML/RAR*α* fusion gene testing was taken by Q-PCR. The time points included pretreatment, CR, every month during consolidation, every three months during maintenance treatments, and every 6 months for at least 2 years. Once PML/RAR*α* fusion turned from negative to positive, the patients would be given ATO for salvage therapy, and if the patients reachieved CR (CR_2_) through the salvage therapy, they would receive consolidation and maintenance chemotherapy mentioned above again and accept a regular monitoring of their PML/RAR*α* fusion gene.

During treatment, patients accepted a regular monitoring of their blood routine, blood coagulation, hepatic and renal function, and electrocardiogram. And changes of their skin, mucous membranes, and nervous, digestive, respiratory, and cardiovascular systems were observed at the same time. Toxic effects were graded according to the National Cancer Institute's Common Toxicity Criteria.

### 2.5. Response Definition and Statistical Consideration

Complete remission (CR) was defined according to conventional criteria, including cellular BM blasts and abnormal promyelocytes < 5%, absolute neutrophil count ≥ 1.5 × 10^9^/L, hemoglobin ≥ 100 g/L, and platelet count ≥ 100 × 10^9^/L [8]. Molecular CR was defined as a negative BM PCR result at sensitivity of 10^−4^ [9]. Clinical recurrence was defined as the presence of ≥5% blasts, or abnormal promyelocytes in the BM, or the appearance of leukemic cells in peripheral blood, or abnormal promyelocytes in cerebrospinal fluid (CSF). Diagnosis of CNS involvement was established in accordance with the symptoms, following lumbar puncture, and morphological, molecular, and/or immunohistochemical analysis of cells CSF. Disease-free survival (DFS) was defined as the time from achieving CR to last follow-up or any event (relapse or death). Overall survival (OS) was defined as the time from initiating treatment to last follow-up of the date of death as a result of any cause. The date of the last follow-up was June 2013.

### 2.6. Statistical Analysis

Statistical analysis was performed by using software SPSS13.0. Rate comparison between two groups was performed by means of *X* ^2^ test. Mann-Whitney test was used for median comparison, and Kaplan-Meier survival curve was used for survival rate analysis. Cumulative incidence of relapse was conducted by using a competing risk method. Results were considered significant at *P* value ≤ 0.05.

## 3. Results

### 3.1. Patient Characteristics

A total of 98 newly diagnosed APL patients who entered CR and accepted postremission therapy were enrolled sequentially in this retrospective study, with a mean ± SD age of 33 ± 8.1 years (range 13–65 years). All of them were treated in QiLu Hospital of Shandong University from January 2003 to December 2008. The date of the last follow-up was June 2013. Except one patient who relapsed and died in experimental group and two who relapsed and died in control group, 95 patients were followed up more than five years. Patients' characteristics including age, sex, and Sanz score show no significant difference between the two groups, as shown in Table 1.

### 3.2. Efficacy Assessment

**Figure 2:**
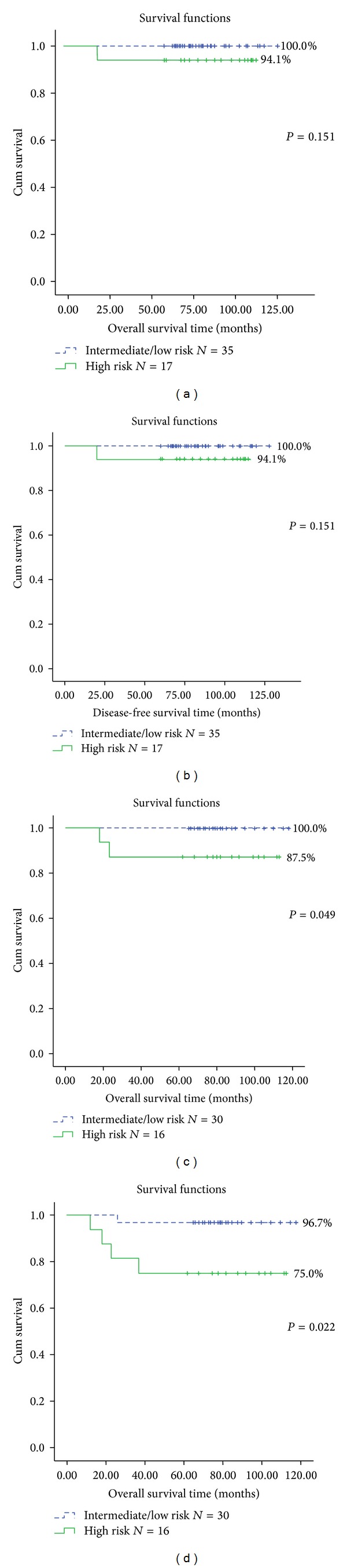
Kaplan-Meier analysis of overall survival (OS) curve and disease-free survival (DFS) curve according to risk in different groups. (a) OS of the experimental group according to risk stratification; (b) DFS of the experimental group according to risk stratification; (c) OS of the control group according to risk stratification; (d) DFS of the control group according to risk stratification.

In experimental group of 52 patients, one patient, who belonged to the high-risk subgroup, relapsed and died in 20 months after diagnose, and the other 51 patients were followed up more than 5 years. The Kaplan-Meier OS and DFS survival curves of the intermediate/low-risk and high-risk subgroups are shown in Figures 2(a) and 2(b). During the median 81.5-month follow-up period (range, 20–128 months), the estimates of OS and DFS were 100% and 94.1%, respectively. There were no statistically significant differences in the estimated OS curves (*P* = 0.151) and DFS curves (*P* = 0.151) between the high-risk and intermediate/low-risk subgroups.

In control group, five patients relapsed at 12, 18, 23, 26, and 37 months after diagnosis. Among them, only one belongs to intermediate-risk subgroup, while the other four all belong to high-risk subgroup. Two of them relapsed in central nervous system (CNS). After salvage therapy, the relapsing one in intermediate-risk subgroup and two in high-risk subgroup reachieved complete remission (CR_2_), while the remaining two relapsing patients in high-risk subgroup died of intracranial hemorrhage and serious infections at 18 and 23 months, respectively. The Kaplan-Meier OS and DFS survival curves of the intermediate/low-risk and high-risk subgroups are shown in Figures 2(c) and 2(d). During the median 80-month follow-up period (range, 18–118 months), the estimates of OS were 100% and 87.5% for the intermediate/low-risk and high-risk subgroups, with significant difference (*P* = 0.049). The estimates of DFS were 96.7% and 75%, also with significant difference (*P* = 0.022). The cumulative incidence of relapse (CIR) in high-risk group was significantly higher than those in intermediate/low-risk groups (*P* = 0.022).

**Figure 3:**
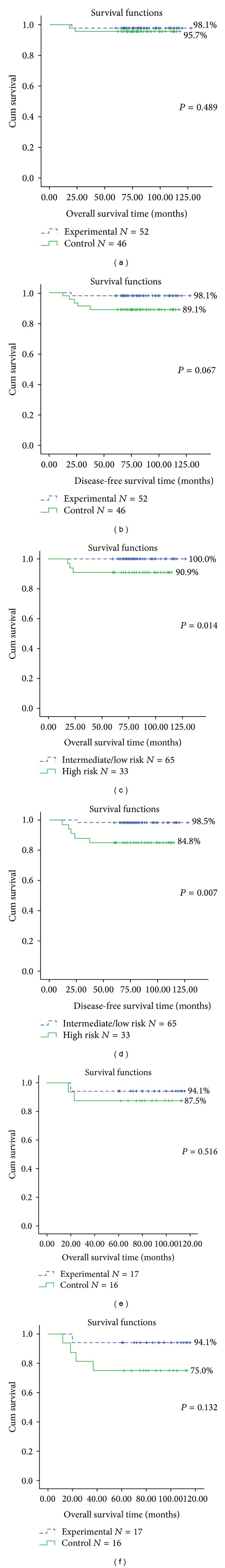
Kaplan-Meier analysis of overall survival (OS) curve and disease-free survival (DFS) curve according to risk in different groups. (a) OS of all patients according to different maintenance therapy; (b) DFS of all patients according to different maintenance therapy; (c) OS of all patients according to risk stratification; (d) DFS of all patients according to risk stratification; (e) OS of high-risk patients according to different maintenance therapy; (f) DFS of high-risk patients according to different maintenance therapy.

The five-year OS and DFS showed no significant difference between experimental group and control group (*P* = 0.489 and 0.067, resp.) (Figures 3(a) and 3(b)). When all 98 patients were grouped according to prognostic risk factors, the high-risk group showed higher relapse and lower DFS than intermediate/low-risk groups (*P* = 0.007) (Figure 3(d)) and the OS also showed significant difference between two groups (*P* = 0.014) (Figure 3(c)), while in high-risk group, the relapse rate showed no significance between two parts of patients using different maintenance therapies.

### 3.3. Effect on PML/RAR*α* Fusion Gene of Different Maintenance Chemotherapy

Before treatment, the test results of PML/RAR*α* fusion gene in 98 patients were all positive. After remission induction and consolidation chemotherapy, the PML/RAR*α* fusion gene of all patients changed to be negative. All 6 relapsing patients had detectable PML/RAR*α* transcript levels before hematologic relapse or CNS relapse. The fusion gene testing results in those patients in persistent remission all kept negative to the end of follow-up.

### 3.4. Toxic Effects

As shown in Table 2, neutropenia is common in experimental group, and all of the symptoms happened after standard chemotherapy was taken in designed sequence during maintenance therapy. When bone marrow suppression finished, all patients recovered rapidly and proceeded to complete the maintenance course without modifying the regimen. RIF shows no hepatotoxicity when it was taken at maintenance therapeutic dose.

The rate of treatment-related liver damage in control group was significantly higher than in experimental group (32.61% and 11.54%, *P* = 0.0112). In experimental group, five cases occurred after chemotherapy and one case occurred after administration of RIF; all of them recovered after the regimens were over without modifying the regimens. In control group, 15 patients suffered from liver damage because of 6-MP, and 3 of them had to experience forced drug withdrawal because of grade 3/4 liver damage.

Oral ulcers were also significantly higher in control group (*P* = 0.0067), which could be overcome without drug withdrawal; however, the oral pain affected eating in different degree, which lowers the quality of life.

Other side effects, such as headache and gastrointestinal symptom, were tolerable and easily overcome in two groups.

## 4. Discussion

Although APL is now a curable disease, the relapse rate remains relatively high in patients who have survived remission, especially in high-risk patients. This has also been proved by our long-term follow-up data, in which all patients with high risk showed significant higher relapse rate than those with intermediate/low risk (*P* = 0.007), especially in those using ATRA and low dose chemotherapy as maintenance therapy (*P* = 0.021), while this difference due to prognostic risk factors could be eliminated when another therapeutic regimen including sequential administration of ATRA, RIF, and chemotherapy was adopted. As our data shows, patients using ATRA/RIF/CT maintenance therapy show no difference in the relapse rate in different risk groups (*P* = 0.151). Although in all patients with high risk using ATRA/RIF/CT regimen groups did not show significant lower relapse rate than those with ATRA/CT_low_ regimen (*P* = 0.132) (the reason may be that the sample size is not large enough), we could still find the advantage of ATRA/RIF/CT regimen in elimination of the difference due to prognostic risk factors in study group.

To improve DFS, many measures have been attempted including strengthening consolidation chemotherapy, adding ATO in maintenance therapy [10], or risk-adapted therapeutic strategies [11]. However, as data shows in the LPA99 study, even using risk-adapted therapeutic strategies, the 3-year CIRs in high-risk groups were still 21% [2]. Our data shows that, in patients using ATRA/RIF/CT regimen, the CIRs in high-risk groups were only 5.9%. The reason why this scheme could overcome high-risk factors and eliminate patient differences may belong to the unique role of RIF in treating APL and strengthened chemotherapy. As demonstrated, arsenic trioxide, including ATO and RIF, was able to eliminate leukemic stem cells in APL, while ATRA cannot [12]. Nevertheless, only using ATO/ATRA as maintenance therapy showed higher CNS leukemia rate in some study in China, compared with other international studies [13]. The reason belonged to the reduction of chemistry induced leukocytosis. Some study also found that, for high-risk types, increasing 1-2 course of chemotherapy can greatly reduce the chances of recurrence of APL [14]. All these suggested that inducing differentiation, promoting apoptosis, and cytotoxic killing effect could play different, even complementary, role to reduce recurrence of APL. Our data shows, using this scheme, 98.1% OS and DFS were gained, and the fusion gene testing in 51 patients followed up to 5 years after complete remission was persistently negative. All these support the efficiency of this regimen despite risk stratification.

Relapse analysis showed that the overall CIRs in control group with the ATRA/CT_low_ regimen were 10.9%, while this rate rose to 25% in high-risk subgroup, which is similar to the former reports. It is worth noting that incidence rate of liver dysfunction is up to 32.61% as daily dose of 6-MP was taken 100 mg per day. Patients often had to reduce drug use or accept drug withdrawal with poor tolerance of those adverse reactions. This decreased medication compliance may be an important reason of relapse. Another reason of relapse perhaps is cellular drug resistance induced by leukemic stem cells, which could be eliminated by arsenic trioxide.

Compared with traditional maintenance regimen, this ATRA/RIF/CT regimen showed lower relapse with better tolerance. In our study, although neutropenia was common after using chemotherapy according to the original plan, the whole course of treatment has not been interrupted for the recovery of bone marrow suppression. This neutropenia in some degree is beneficial to reduce the relapse as mentioned above. Besides, RIF shows less hepatotoxicity or liver dysfunction when it is taken at maintenance therapeutic dose (60 mg/kg·d), remarkably lower than the dose in inducing therapy (120 mg/kg·d), which shows gastrointestinal reactions in different degrees [15].

The survival curve analysis showed that the recurrence peak was in 2 years after CR, which indicated that this period was critical to reduce relapse. This is why the treatment cycle was maintained for 2 years. During these 2 years, patients using ATRA/RIF/CT regimen only need to accept 3-cycle chemotherapy in hospital, while other therapies are all taken orally outside the hospital. This regimen could reduce the hospitalization days and costs and get a good efficiency cost ratio. As there was a longer treatment intermittent after one course of treatment, this regimen could keep organism sensitive to chemotherapy drugs and avoid the accumulation of arsenic poisoning. And in that interval, various organ and immune functions of body could be restored to keep the continuity of treatment.

In this study, our repeated sequential therapeutic regimen to APL with ATRA, RIF, and chemotherapy shows better efficacy and less toxicity, especially in high-risk patients. However, our findings should be interpreted with caution because of two limits; one limit is that our study is retrospective and the other is that the study is based on a single-center experience. We need further long-term follow-up studies to explore its curative effect as a more long-term maintenance treatment.

## Figures and Tables

**Table 1:** Patients' characteristics in two groups.

Parameters	Experimental group (*n* = 52)	Control group (*n* = 46)
Gender		
Male	32	26
Female	20	20
Age (years)	14–63	13–65
WBC count (×10^9^/L)		
≤10 × 10^9^/L	35	30
>10 × 10^9^/L	17	16
Platelet count (×10^9^/L)		
>40 × 10^9^/L	18	16
≤40 × 10^9^/L	34	30
Sanz score		
Low risk	10	8
Intermediate risk	25	22
High risk	17	16
Median follow-up time (months)	81.5 (20–128)	80 (18–118)

Sanz score: low risk, WBC ≤ 10 × 10^9^/L and platelets > 40 × 10^9^/L; intermediate risk, WBC ≤ 10 × 10^9^/L and platelets ≤ 40 × 10^9^/L; high risk, WBC > 10 × 10^9^/L.

Induction chemotherapy according to Sanz score: low risk, ATRA; intermediate risk, ATRA + DNR/DA; high risk, ATRA + DA + ATO; consolidation chemotherapy, DA/MA/HA regimen used sequentially in all patients despite their Sanz scores.

**Table 2:** Toxicity profile in two groups.

Parameters	Experimental group (*n* = 52)		Control group (*n* = 46)	
Grade	1-2	3-4	1-2	3-4
Neutropenia	12	40	14	0
Liver damage	6	0	12	3
Renal damage	0	0	0	0
Cardiac damage	0	0	0	0
Gastrointestinal symptom	5	0	4	0
Oral ulcer	0	0	7	0

Neutropenia was higher in experimental group (*P* = 0), while liver damage is higher in control group (*P* = 0.0112).
